# Claims-based algorithms for identifying Medicare beneficiaries at high estimated risk for coronary heart disease events: a cross-sectional study

**DOI:** 10.1186/1472-6963-14-195

**Published:** 2014-04-29

**Authors:** Evan L Thacker, Paul Muntner, Hong Zhao, Monika M Safford, Jeffrey R Curtis, Elizabeth Delzell, Vera Bittner, Todd M Brown, Emily B Levitan

**Affiliations:** 1 Department of Epidemiology, University of Alabama at Birmingham, Birmingham, AL 35294-0022, USA; 2 Department of Health Science, Brigham Young University, Provo, UT, USA; 3 Department of Medicine, University of Alabama at Birmingham, Birmingham, AL, USA

**Keywords:** High risk, Coronary heart disease, Risk stratification, Medicare claims

## Abstract

**Background:**

Databases of medical claims can be valuable resources for cardiovascular research, such as comparative effectiveness and pharmacovigilance studies of cardiovascular medications. However, claims data do not include all of the factors used for risk stratification in clinical care. We sought to develop claims-based algorithms to identify individuals at high estimated risk for coronary heart disease (CHD) events, and to identify uncontrolled low-density lipoprotein (LDL) cholesterol among statin users at high risk for CHD events.

**Methods:**

We conducted a cross-sectional analysis of 6,615 participants ≥66 years old using data from the REasons for Geographic And Racial Differences in Stroke (REGARDS) study baseline visit in 2003–2007 linked to Medicare claims data. Using REGARDS data we defined high risk for CHD events as having a history of CHD, at least 1 risk equivalent, or Framingham CHD risk score >20%. Among statin users at high risk for CHD events we defined uncontrolled LDL cholesterol as LDL cholesterol ≥100 mg/dL. Using Medicare claims-based variables for diagnoses, procedures, and healthcare utilization, we developed algorithms for high CHD event risk and uncontrolled LDL cholesterol.

**Results:**

REGARDS data indicated that 49% of participants were at high risk for CHD events. A claims-based algorithm identified high risk for CHD events with a positive predictive value of 87% (95% CI: 85%, 88%), sensitivity of 69% (95% CI: 67%, 70%), and specificity of 90% (95% CI: 89%, 91%). Among statin users at high risk for CHD events, 30% had LDL cholesterol ≥100 mg/dL. A claims-based algorithm identified LDL cholesterol ≥100 mg/dL with a positive predictive value of 43% (95% CI: 38%, 49%), sensitivity of 19% (95% CI: 15%, 22%), and specificity of 89% (95% CI: 86%, 90%).

**Conclusions:**

Although the sensitivity was low, the high positive predictive value of our algorithm for high risk for CHD events supports the use of claims to identify Medicare beneficiaries at high risk for CHD events.

## Background

National Cholesterol Education Program Adult Treatment Panel III (ATP III) guidelines and the newly released 2013 American College of Cardiology/American Heart Association Guideline on the Treatment of Blood Cholesterol to Reduce Atherosclerotic Cardiovascular Risk in Adults recommend lipid treatment according to estimated risk for future coronary heart disease (CHD) events such as nonfatal myocardial infarction or CHD death [1-3]. Pharmacotherapy initiation is guided by low-density lipoprotein (LDL) cholesterol levels and risk for future CHD or other atherosclerotic events. Risk is assessed by history of CHD, risk equivalents such as stroke and diabetes, risk factors such as hypertension and current smoking, and predicted risk calculated from risk equations including CHD risk factors [1-3]. Despite these guidelines, many people eligible for lipid lowering therapy are untreated or undertreated [4,5].

Novel LDL cholesterol lowering medications are being evaluated in clinical trials [6,7]. If these medications obtain regulatory approval, healthcare claims data could be used for comparative effectiveness and pharmacovigilance studies [8]. Identifying people using medications is feasible with pharmacy claims. Finding a comparison group with a similar CHD risk profile is more challenging. One potential barrier is that claims data do not include clinical or laboratory values that are often used to estimate CHD event risk. Some data show that claims-based algorithms can be useful in identifying high risk groups, for example people at high risk for osteoporotic fracture [9]. However, whether claims-based algorithms can be used to identify individuals at high risk for CHD events is not known.

In addition to identifying high risk groups, it would be of interest in some studies to identify people who may warrant more intensive treatment based on laboratory tests; one such group is individuals with elevated LDL cholesterol levels despite statin treatment. So far there is not much evidence that healthcare claims can be used effectively to estimate laboratory values [10]. Advances in this area could increase the value of healthcare claims for comparative effectiveness and pharmacovigilance research.

In this paper we describe the development of claims-based algorithms to identify individuals at high risk for CHD events according to ATP III guidelines, using Medicare claims data on diagnoses, procedures, and healthcare utilization. We expanded upon existing claims-based definitions of specific cardiovascular conditions and procedures [11-19] by bringing them into a broader framework with the goal of identifying the more general concept of “high risk.” We also describe claims-based algorithms to identify uncontrolled LDL cholesterol according to ATP III guidelines among statin users at high risk for CHD events. In evaluating these algorithms we considered positive predictive value as the most important measure of model performance because our aim was to identify high-risk groups and exclude lower-risk individuals.

## Methods

### Design, setting, and participants

**Figure 1:**
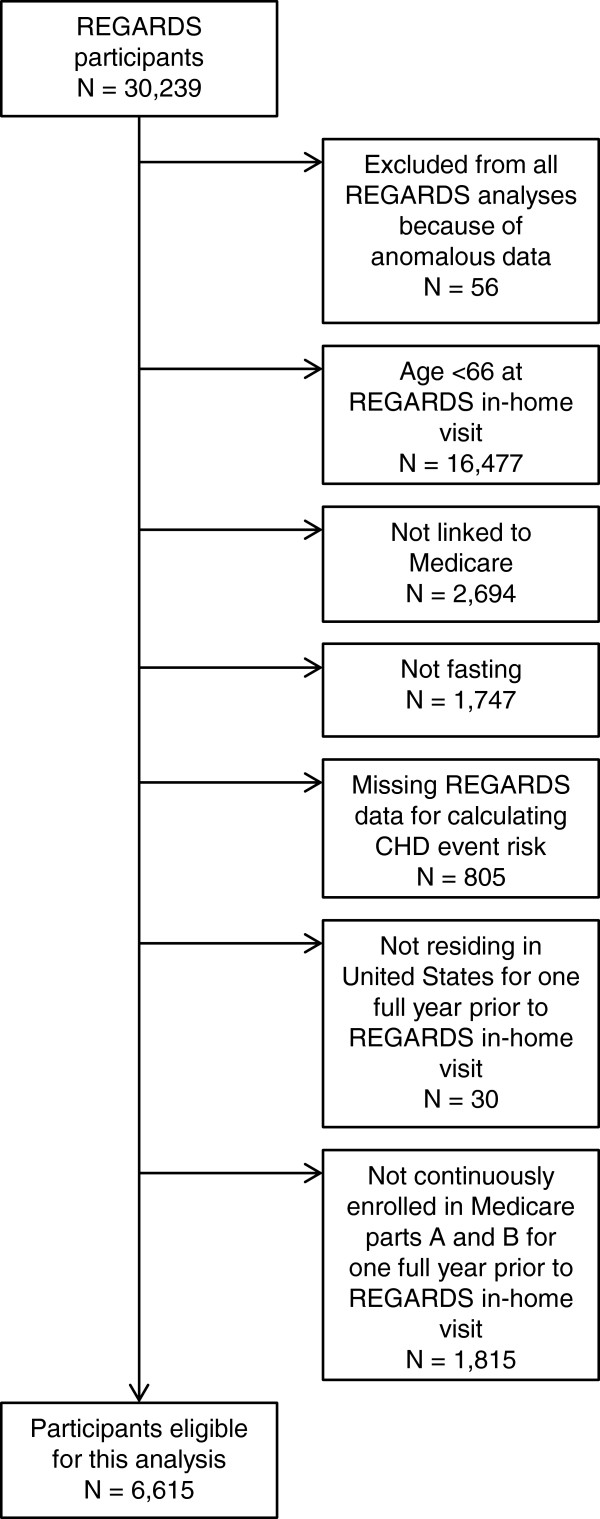
Participant flowchart.

We conducted a cross-sectional analysis of baseline data from REasons for Geographic And Racial Differences in Stroke (REGARDS) study participants linked to Medicare data. REGARDS is a population-based cohort of 30,239 adults ≥45 years of age enrolled in 2003–2007 in the continental United States [20]. Linkage of REGARDS data with Medicare enrollment data was based on social security number, which is a unique identifier that was required to match exactly on all digits; sex, which was required to match; and birth date, which was required to match on year and month, year and day, or month and day allowing a difference of one year. We included 6,615 REGARDS participants who provided study data free of anomalies such as missing all baseline data collection forms, were ≥66 years of age at their REGARDS in-home visit, linked to Medicare, provided a fasting blood sample, had complete REGARDS data for calculating CHD event risk according to ATP III guidelines (described below), and had been living in the United States, continuously enrolled in Medicare parts A and B, but not in a Medicare Advantage plan, for at least one year immediately prior to their REGARDS in-home visit (Figure 1). This research was conducted in accordance with the Declaration of Helsinki. Institutional review boards of the collaborating institutions (University of Alabama at Birmingham, University of Vermont, Wake Forest University, and University of Cincinnati) approved the REGARDS protocol. Participants gave informed consent. The REGARDS-Medicare linkage was approved by the institutional review board at the University of Alabama at Birmingham.

### REGARDS variables

REGARDS baseline data collection included a structured telephone interview and an in-home visit. The telephone interview included questions about demographics and medical history. The in-home visit included two blood pressure measurements, an electrocardiogram, blood sample collection, and a medication inventory to assess current medication use including statins (see Text, Additional file 1).

Using REGARDS data we categorized each participant’s CHD event risk according to the ATP III guidelines 2004 update [1,2]. High risk for CHD events was defined as (1) having a history of CHD, including myocardial infarction (MI) or coronary revascularization; (2) having a history of least one of the following risk equivalents: peripheral arterial disease, abdominal aortic aneurysm, carotid artery disease, stroke, or diabetes; or (3) in the absence of CHD or risk equivalents, having ≥2 CHD risk factors with Framingham 10-year CHD risk score >20%. CHD risk factors included age ≥45 years (men) or ≥ 55 years (women), family history of premature MI, current smoking, hypertension, and high-density lipoprotein (HDL) cholesterol <40 mg/dL. HDL cholesterol ≥60 mg/dL reduced the risk factor count by one. Framingham 10-year CHD risk score >20% was defined using age, sex, total cholesterol, HDL cholesterol, current smoking, and systolic blood pressure [1]. Very high risk for CHD events was defined as having CHD and at least one of the following: acute MI in the prior year, diabetes, current smoking, or metabolic syndrome.

Using the above definitions and REGARDS study data, we defined the presence of two high risk conditions among REGARDS-Medicare linked participants (N = 6,615):

● Condition 1: High risk for CHD events

● Condition 2: Very high risk for CHD events

We defined a third high risk condition among REGARDS-Medicare linked participants who did not have a history of CHD or risk equivalents (N = 3,720):

● Condition 3: Framingham 10-year CHD risk score >20%

Also using REGARDS study data, we defined the presence of uncontrolled LDL cholesterol, using two cut-points, among REGARDS-Medicare linked participants at high risk for CHD events who were using statins according to the REGARDS in-home visit medication inventory (N = 1,583):

● Condition 4: LDL cholesterol ≥100 mg/dL

● Condition 5: LDL cholesterol ≥70 mg/dL

### Medicare Variables

To develop claims-based algorithms we used Medicare data from January 2000 to the date of the REGARDS in-home visit (2003 to 2007). Mean time from Medicare enrollment to the REGARDS in-home visit was 4.6 years (range: 1.1 to 7.8 years). We identified Medicare variables using two approaches. First, we pre-specified 25 variables describing demographics, diagnoses, and healthcare utilization indicators that we believed would be associated with CHD risk:

● **Demographic variables** including age, sex, race, Medicaid eligibility, area-level income, and geographic region.

● **Diagnosis variables** including claims-based evidence of tobacco use, history of hyperlipidemia, history of hypertension, history of diabetes, acute myocardial infarction, coronary revascularization, history of coronary heart disease, history of stroke, history of abdominal aortic aneurysm, history of peripheral arterial disease, and history of carotid artery disease.

● **Health care utilization variables** including cardiologist care, endocrinologist care, neurologist care, number of evaluation and management visits, hospitalization for any cause, cardiac stress test, echocardiogram, and electrocardiogram.

Several pre-specified variables were based on published claims-based definitions (see Text, Additional file 1) [11-19]. The same 25 pre-specified variables were used in algorithms for all conditions of interest. Second, through a data mining procedure we identified additional Medicare variables, including diagnosis and procedure codes. The data mining procedure was adapted from a previously described algorithm for high-dimensional propensity scores (for detail see Text, Additional file 1) [21]. The procedure had four steps: (1) identify diagnosis and procedure codes appearing in REGARDS participants’ linked Medicare claims data, (2) calculate the prevalence of each code, (3) calculate the odds ratio of each code with high risk as defined using REGARDS data, and (4) rank the codes as a function of their prevalence and their odds ratio with high risk, and select the highest-ranked codes, excluding collinear variables. The data mining variables differed for each condition. We did not use Medicare Part D pharmacy claims data because few participants had Part D coverage prior to the REGARDS in-home visit.

### Statistical analysis

We used logistic regression and Medicare claims-based variables to develop algorithms identifying each of three high risk conditions and uncontrolled LDL cholesterol defined in REGARDS data. The analysis is described below for Condition 1, high risk for CHD events. An identical approach was used for the other conditions. We calculated beta coefficients and standard errors for high risk associated with each pre-specified Medicare variable from a multivariable logistic regression model. We included interaction terms for sex with other variables if they had a P value <0.1. We calculated the predicted probability of being at high risk for each participant from the beta coefficients, and plotted distributions of predicted probabilities for people at high risk and for people not at high risk according to REGARDS data. We calculated sensitivity, specificity, positive predictive value (PPV), and negative predictive value (NPV) across the range of predicted probability thresholds from 0 to 1, and we calculated a c-statistic. We calculated 95% confidence intervals for the model performance characteristics by bootstrapping. To report model performance characteristics in tables we chose the predicted probability threshold that resulted in 90% specificity before correcting for optimism (see below). We built a second model adding variables from the data mining procedure. We corrected model performance characteristics for optimism using bootstrap resampling, which has been recommended as a better method for internal validation than a split-sample approach [22]. We cross-classified participants by model-predicted and observed high risk status to compare characteristics of true positives, false positives, false negatives, and true negatives.

We conducted four sensitivity analyses. First, for identifying high risk, we assigned a predicted probability of 1 for each participant who had evidence in their claims of a history of CHD, peripheral arterial disease, abdominal aortic aneurysm, carotid artery disease, stroke, or diabetes; and assigned a predicted probability of 0 otherwise. Second, for identifying high risk, we assigned a predicted probability of 1 as in the first sensitivity analysis; and assigned a predicted probability based on a logistic regression model otherwise. Third, for identifying very high risk, we assigned a predicted probability of 1 for each participant who had evidence in their claims of a history of CHD and either acute MI in the prior year or diabetes; and assigned a predicted probability based on a logistic regression model otherwise. Fourth, for all five conditions, we used claims data for only one year prior to the REGARDS in-home visit to define pre-specified and data mining Medicare variables, instead of using all available claims.

We used SAS software version 9.3 (Cary, NC) for all statistical analyses.

## Results

### High risk for CHD events

**Figure 2:**
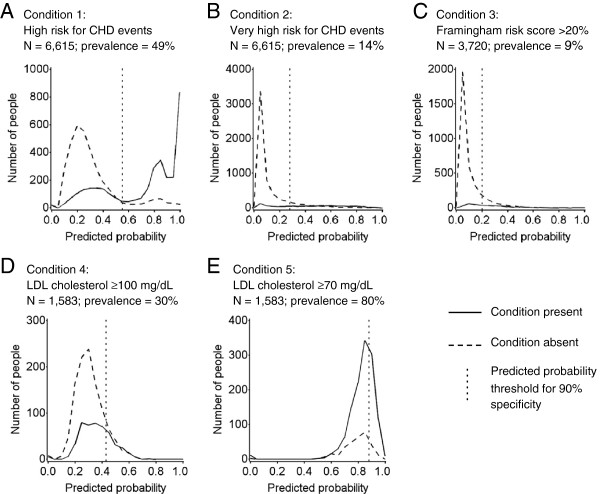
**Distributions of predicted probabilities of having a high risk condition or an uncontrolled LDL cholesterol condition (estimated by Medicare data) by observed presence or absence of the condition (REGARDS study data).** Panel **A** shows Condition 1, high risk for CHD events, among all eligible participants. Panel **B** shows Condition 2, very high risk for CHD events, among all eligible participants. Panel **C** shows Condition 3, Framingham CHD risk score >20%, among eligible participants without a history of CHD or risk equivalents. Panel **D** shows Condition 4, LDL cholesterol ≥100 mg/dL, and Panel **E** shows Condition 5, LDL cholesterol ≥70 mg/dL, among eligible participants at high risk for CHD events who were using statins according to the REGARDS in-home visit medication inventory. In each panel the solid curve represents the distribution of predicted probabilities of having the condition among those observed to have the condition in REGARDS, the dashed curve represents the distribution of predicted probabilities of having the condition among those not observed to have the condition in REGARDS, and the vertical dotted line represents the predicted probability threshold corresponding to 90% specificity for identifying the condition. All results are from models using pre-specified Medicare variables only. Results from models using pre-specified variables plus data mining variables were similar (data not shown).

Among 6,615 REGARDS participants, 49% were at high risk for CHD events based on REGARDS data. High risk participants were more likely to be men, have less education, be using statins, have lower cholesterol, and higher levels of other cardiovascular risk factors including hypertension and metabolic syndrome, compared with participants not at high risk (Table 1). Predicted probabilities using pre-specified variables tended to be higher for participants at high risk and lower for participants not at high risk (Figure 2, Panel A). In the model that included pre-specified variables, a predicted probability threshold of 0.55 yielded a PPV of 87% (95% CI: 85%, 88%) for identifying high risk for CHD, and a sensitivity of 69% (95% CI: 67%, 70%); results were similar after adding data mining variables (Table 2 and see Additional file 1: Figure S1, Panel A). High risk participants not identified by the algorithm (false negatives) were less likely to be men, be using statins, have metabolic syndrome, diabetes, and CHD, and had higher LDL cholesterol, higher blood pressure, and lower blood glucose, compared with high risk participants correctly identified by the algorithm (true positives) (Table 3). Non-high risk participants identified as high risk by the algorithm (false positives) were less likely to be using statins, to have metabolic syndrome, and had higher total and LDL cholesterol but lower triglycerides, compared with true positives.

**Table 1:** Participant characteristics in REGARDS study data by CHD risk category*

**Characteristic†**	**Overall**	**High risk for CHD events**	**Not high risk for CHD events**
	**N = 6,615**	**N = 3,216**	**N = 3,399**
Age, y	73.2 (5.6)	73.9 (5.6)	72.7 (5.5)
Male	49.6	60.0	39.7
Black	30.4	32.9	28.1
Income < $35,000/y	56.7	58.7	54.6
Education ≤ High school graduate	40.6	45.4	36.2
Current use of statins	37.6	49.2	26.6
Total cholesterol, mg/dL	187.0 (38.9)	177.8 (38.5)	195.6 (37.3)
LDL cholesterol, mg/dL	109.8 (34.0)	103.4 (33.8)	115.9 (33.1)
LDL cholesterol among statin non-users, mg/dL	120.4 (33.9)	117.3 (35.4)	122.5 (32.8)
HDL cholesterol, mg/dL	52.1 (16.4)	47.6 (15.1)	56.3 (16.5)
Triglycerides, mg/dL	125.3 (61.5)	133.9 (66.0)	117.1 (55.8)
Family history of MI	19.0	20.9	17.4
Current cigarette smoking	8.9	11.0	6.9
Body mass index, kg/m^2^	28.1 (5.4)	28.9 (5.4)	27.4 (5.2)
Systolic blood pressure, mm Hg	130.3 (16.6)	133.2 (17.5)	127.5 (15.1)
Diastolic blood pressure, mm Hg	75.3 (9.4)	75.6 (9.8)	75.1 (8.9)
Blood glucose, mg/dL	101.4 (27.9)	110.7 (36.3)	92.7 (10.5)
C-reactive protein, mg/L	4.5 (8.5)	5.0 (9.4)	4.0 (7.5)
Hypertension	64.7	75.2	54.8
Metabolic syndrome	39.0	54.4	24.5
Coronary heart disease	24.6	50.6	0.0‡
Acute MI in prior year	1.3	2.8	0.0‡
Peripheral arterial disease	2.6	5.3	0.0‡
Abdominal aortic aneurysm	1.6	3.3	0.0‡
Carotid artery disease	3.2	6.7	0.0‡
Stroke	7.6	15.7	0.0‡
Diabetes	20.8	42.8	0.0‡

*Abbreviations*: *HDL* high density lipoprotein, *LDL* low density lipoprotein *MI* myocardial infarction.

*REGARDS participants included in this table are those who were linked to Medicare data and met all eligibility criteria for this analysis (see Figure 1).

†Numbers are column percentages or means (standard deviations). Income was missing for 914 participants, education for 4, body mass index for 16, and C-reactive protein for 164.

‡By definition, participants not at high risk for CHD events did not have a history of CHD or risk equivalents according to REGARDS study data.

**Table 2:** Test characteristics of Medicare claims-based models for identifying high risk conditions or uncontrolled LDL cholesterol conditions defined in REGARDS study data

Condition and model*	**N**	**Prevalence of condition**	**Predicted probability threshold**^**†**^	**Sensitivity (95% CI)**^**‡**^	**Specificity (95% CI)**^**‡**^	**PPV (95% CI)**^**‡**^	**NPV (95% CI)**^**‡**^	**C statistic (95% CI)**^**‡**^
*Among all eligible participants*								
Condition 1: High risk for CHD events	6,615	49%						
Pre-specified			0.55	0.69 (0.67, 0.70)	0.90 (0.89, 0.91)	0.87 (0.85, 0.88)	0.75 (0.73, 0.77)	0.86 (0.85, 0.86)
Pre-specified + data mining			0.52	0.71 (0.69, 0.72)	0.89 (0.88, 0.90)	0.86 (0.84, 0.87)	0.76 (0.74, 0.77)	0.87 (0.86, 0.88)
Condition 2: Very high risk for CHD events	6,615	14%						
Pre-specified			0.28	0.63 (0.59, 0.66)	0.90 (0.89, 0.90)	0.52 (0.49, 0.54)	0.94 (0.93, 0.95)	0.84 (0.83, 0.86)
Pre-specified + data mining			0.26	0.66 (0.63, 0.70)	0.90 (0.89, 0.90)	0.52 (0.49, 0.55)	0.94 (0.93, 0.94)	0.86 (0.84, 0.87)
*Among eligible participants without history of CHD or risk equivalents*								
Condition 3: Framingham risk score >20%	3,720	9%						
Pre-specified			0.20	0.47 (0.43, 0.54)	0.90 (0.89, 0.91)	0.31 (0.27, 0.36)	0.95 (0.94, 0.96)	0.82 (0.80, 0.84)
Pre-specified + data mining			0.20	0.46 (0.40, 0.52)	0.90 (0.89, 0.91)	0.31 (0.27, 0.35)	0.95 (0.94, 0.95)	0.81 (0.79, 0.84)
*Among eligible participants at high risk for CHD events who were using statins*								
Condition 4: LDL cholesterol ≥100 mg/dL	1,583	30%						
Pre-specified			0.43	0.19 (0.15, 0.22)	0.89 (0.86, 0.90)	0.43 (0.38, 0.49)	0.72 (0.70, 0.74)	0.60 (0.57, 0.62)
Pre-specified + data mining			0.45	0.20 (0.15,0.24)	0.88 (0.86, 0.89)	0.43 (0.39, 0.48)	0.71 (0.69, 0.73)	0.62 (0.60, 0.65)
Condition 5: LDL cholesterol ≥70 mg/dL	1,583	80%						
Pre-specified			0.88	0.19 (0.17, 0.23)	0.86 (0.81, 0.90)	0.86 (0.83, 0.89)	0.21 (0.19, 0.23)	0.59 (0.56, 0.62)
Pre-specified + data mining			0.88	0.25 (0.23, 0.27)	0.85 (0.81, 0.88)	0.91 (0.85, 0.91)	0.23 (0.20, 0.24)	0.60 (0.57, 0.63)

*Abbreviations*: *NPV* negative predictive value, *PPV* positive predictive value.

*Pre-specified Medicare variables: age, sex, race, Medicaid eligible, area-level income, geographic region, evidence of tobacco use, history of hyperlipidemia, history of hypertension, history of diabetes, acute MI, coronary revascularization, history of CHD, history of stroke, history of abdominal aortic aneurysm, history of peripheral arterial disease, history of carotid artery disease, cardiologist care, endocrinologist care, neurologist care, number of evaluation and management visits, hospitalization for any cause, cardiac stress test, echocardiogram, electrocardiogram. For pre-specified variable definitions and a description of the methods used to obtain variables through a data mining procedure see Additional file 1.

^†^For each model, test characteristics are reported for the predicted probability threshold corresponding to an uncorrected specificity of 0.90.

^‡^Corrected for optimism using bootstrap resampling [22].

**Table 3:** Participant characteristics from REGARDS data by observed (REGARDS) and model-predicted (Medicare) high risk for CHD events, from the model with pre-specified Medicare variables only

	**Model-predicted high risk (Medicare)**		**Model-predicted not high risk (Medicare)**	
Characteristic*	**Observed high risk (REGARDS) (true positives) N = 2,217**	**Observed not high risk (REGARDS) (false positives) N = 343**	**Observed high risk (REGARDS) (false negatives) N = 999**	**Observed not high risk (REGARDS) (true negatives) N = 3,056**
Age, y	74.0 (5.7)	74.6 (6.0)	73.6 (5.5)	72.4 (5.3)
Male	63.9	57.4	51.3	37.7
Black	34.6	35.0	28.9	27.3
Income < $35,000/y	58.5	58.0	59.3	54.3
Education ≤ High school graduate	45.7	42	44.6	35.5
Current use of statins	57.5	38.2	30.9	25.3
Total cholesterol, mg/dL	172.0 (37.2)	182.9 (38.6)	190.8 (38.3)	197.0 (36.9)
LDL cholesterol, mg/dL	98.7 (32.3)	106.0 (33.9)	114.0 (34.5)	117.0 (32.9)
LDL cholesterol among statin non-users, mg/dL	113.3 (35.1)	115.3 (34.8)	122.7 (35.1)	123.1 (32.5)
HDL cholesterol, mg/dL	47.0 (14.4)	53.9 (17.0)	48.9 (16.5)	56.6 (16.5)
Triglycerides, mg/dL	131.2 (65.9)	114.6 (58.1)	139.9 (65.7)	117.4 (55.5)
Family history of MI	21.9	19.0	19.0	17.2
Current cigarette smoking	9.2	6.1	15.0	7.0
Body mass index, kg/m^2^	29.2 (5.5)	28.4 (5.6)	28.1 (5.2)	27.3 (5.2)
Systolic blood pressure, mm Hg	132.7 (17.6)	129.3 (15.0)	134.2 (17.4)	127.3 (15.2)
Diastolic blood pressure, mm Hg	74.9 (9.7)	75.1 (9.0)	77 (9.9)	75.1 (8.9)
Blood glucose, mg/dL	114.2 (38.4)	95.6 (12.6)	102.9 (29.8)	92.4 (10.1)
C-reactive protein, mg/L	4.9 (9.3)	4.4 (10.7)	5.2 (9.6)	3.9 (7.1)
Hypertension	76.0	66.5	73.5	53.5
Metabolic syndrome	57.8	32.9	46.7	23.6
Diabetes	54.0	0.0†	18.0	0.0†
Coronary heart disease	55.3	0.0†	40.1	0.0†
Acute MI in prior year	‡	0.0†	‡	0.0†
Peripheral arterial disease	5.5	0.0†	4.8	0.0†
Abdominal aortic aneurysm	4.0	0.0†	1.9	0.0†
Carotid artery disease	7.7	0.0†	4.4	0.0†
Stroke	16.2	0.0†	14.5	0.0†

*Abbreviations*: HDL high density lipoprotein, LDL low density lipoprotein, MI myocardial infarction.

*Numbers are column percentages or means (standard deviations). Income was missing for 914 participants, education for 4, body mass index for 16, and C-reactive protein for 164.

†By definition, participants not at high risk for CHD events did not have a history of CHD or risk equivalents according to REGARDS study data.

‡The Centers for Medicare and Medicaid Services (CMS) requires the figure be redacted because the cell contained fewer than 11 participants, or would allow a number fewer than 11 participants to be deduced in another cell.

### Very high risk for CHD events

Among 6,615 participants, 14% were at very high risk for CHD events based on REGARDS data. Predicted probabilities were uniformly distributed for participants at very high risk and tended to be low for participants not at very high risk (Figure 2, Panel B). In the model using pre-specified variables, a predicted probability threshold of 0.28 yielded a PPV of 52% (95% CI: 49%, 54%) for identifying very high risk for CHD events and a sensitivity of 63% (95% CI: 59%, 66%); results were similar after adding data mining variables (Table 2 and see Additional file 1: Figure S1, Panel B). Participant characteristics by observed and model-predicted very high risk status are in Additional file 1: Table S1.

### Framingham CHD risk score >20%

Among 3,720 participants who did not have a history of CHD or risk equivalents according to REGARDS data, 9% had Framingham 10-year CHD risk scores >20%. Predicted probabilities were uniformly distributed for participants with risk scores >20% and tended to be low for participants with risk scores ≤20% (Figure 2, Panel C). In the model using pre-specified variables, a predicted probability threshold of 0.20 yielded a PPV of 31% (95% CI: 27%, 36%) for identifying Framingham CHD risk score >20% and a sensitivity of 47% (95% CI: 43%, 54%); results were similar after adding data mining variables (Table 2 and see Additional file 1: Figure S1, Panel C). Participant characteristics by observed and model-predicted Framingham CHD risk score >20% status are in Additional file 1: Table S2.

### Uncontrolled LDL cholesterol among statin users at high risk for CHD events

Among 1,583 participants at high risk for CHD events who were using statins according to the REGARDS medication inventory, 30% had LDL cholesterol ≥100 mg/dL, and 80% had LDL cholesterol ≥70 mg/dL. The predicted probability distributions overlapped for participants with and without uncontrolled LDL cholesterol (Figure 2). In the model using pre-specified variables, a predicted probability threshold of 0.43 yielded a PPV of 43% (95% CI: 38%, 49%) for identifying LDL cholesterol ≥100 and a sensitivity of 19% (95% CI: 15%, 22%) (Table 2 and see Additional file 1: Figure S2, Panel A). For identifying LDL cholesterol ≥70 the PPV was 86% (95% CI: 83%, 89%) in the model using pre-specified variables, and increased to 91% (95% CI: 85%, 91%) when adding data mining variables (Table 2 and Additional file 1: Figure S2, Panel B). Participant characteristics by observed and model-predicted uncontrolled LDL cholesterol status are in Additional file 1: Tables S3 and S4.

### Model parameters

Beta coefficients and standard errors for models using pre-specified Medicare variables are in Additional file 1: Table S5.

### Sensitivity analyses

When we assigned a predicted probability of 1 for participants who met a pre-specified claims-based definition of high risk for CHD events, and a predicted probability of 0 otherwise, the PPV was 80% (95% CI: 79%, 81%) and sensitivity was 75% (95% CI: 73%, 77%). Model characteristics were similar when we assigned a predicted probability of 1 for participants who met a pre-specified claims-based definition of high risk for CHD events, and assigned model-based predicted probabilities otherwise. When we assigned a predicted probability of 1 for participants who met a pre-specified claims-based definition of very high risk for CHD events, and assigned model-based predicted probabilities otherwise, the PPV was 51% (95% CI: 48%, 54%) and sensitivity was 63% (95% CI: 58%, 66%) (see Additional file 1: Table S6, and Additional file 1: Figure S3).

When we used only one year of Medicare claims data prior to the REGARDS in-home visit, the sensitivity decreased for identifying high risk for CHD events, very high risk for CHD events, and Framingham 10-year CHD risk score >20% (see Additional file 1: Table S7). Results for uncontrolled LDL cholesterol among statin users at high risk for CHD events were similar to the main results when we used only one year of Medicare claims data (see Additional file 1: Table S8).

## Discussion

In this population of REGARDS study participants, an algorithm using 25 pre-specified Medicare claims variables had a PPV of 87% for identifying people at high risk for CHD events. Additional claims variables identified through data mining did not substantially improve the algorithm performance. The high PPV of our algorithm supports the use of claims to identify Medicare beneficiaries at high risk for CHD events. Our algorithm could be applied in comparative effectiveness or pharmacovigilance studies of the outcomes of cardiovascular medication use among Medicare beneficiaries. For example, if novel LDL cholesterol lowering drugs currently in development [6,7] come to market, Medicare may be a setting in which to evaluate the effectiveness and safety of these drugs. Comparison groups in such studies should have comparable proportions of people at high risk for CHD events, and our algorithm could be used to identify appropriate comparison cohorts. However, the algorithm had a sensitivity of 69% and missed 31% of participants at high risk for CHD events. Along with this low sensitivity, the group identified was not representative of all participants at high risk for CHD events. People at high risk for CHD events whom our algorithm missed were less likely to have CHD and diabetes and to be using statins, and had higher lipid levels and blood pressure compared with people at high risk for CHD events whom the algorithm correctly identified. This pattern is consistent with claims data being more sensitive for identifying people with diagnosed conditions than for identifying people with abnormal laboratory values. Therefore, future use of this algorithm to identify people at high risk for CHD events should be accompanied by careful consideration of generalizability.

Our algorithms did not perform as well for identifying those at very high risk for CHD events and those without CHD or risk equivalents but with a Framingham 10-year CHD risk score >20%. Very high risk for CHD events and Framingham 10-year CHD risk score >20% had low prevalence. Because PPV depends on the prevalence of the condition, it is not surprising that PPVs for identifying these subgroups were low. A prior study has reported claims data on diagnoses, procedures, and healthcare utilization may not be good proxies for clinical and laboratory values [10]. The current analysis extends this prior finding and indicates claims data have limited usefulness for identifying individuals with a Framingham 10-year CHD risk score >20%.

Among participants at high risk for CHD events who were using statins, algorithms identified those with LDL cholesterol ≥100 mg/dL with a PPV of 43% using pre-specified variables. As expected, the PPV for identifying LDL cholesterol ≥70 mg/dL was higher (86%), due to higher prevalence of the condition. However, true positives, false positives, and false negatives differed on several characteristics. The gain in PPV by using a claims-based algorithm to identify individuals with LDL cholesterol ≥70 mg/dL may not outweigh the potential loss in representativeness as compared with all statin users at high risk for CHD events.

In comparison with using all available Medicare claims prior to the REGARDS baseline study visit for each participant, limiting Medicare claims to the one year period prior to REGARDS baseline decreased the sensitivity for identifying participants at high estimated risk for CHD events. Evidently some Medicare beneficiaries with a history of cardiovascular conditions or risk factors do not have sufficient evidence of those conditions or risk factors in recent diagnosis and procedure codes. Therefore, studies in which participants’ Medicare history is limited to a certain time period may tend toward underestimating the prevalence of high risk for CHD events.

To our knowledge, there are no prior reports of claims-based algorithms for identifying high risk for CHD events according to the ATP III definition. Published claims-based definitions of several cardiovascular conditions and procedures are available, and we incorporated these into our algorithms as pre-specified variables [11-19]. In this study we attempted to go beyond specific disease diagnoses to identify a more broadly-defined group at high risk for CHD events according to ATP III guidelines. As in the ATP III guidelines, the newly published cholesterol treatment guidelines recommend that treatment decisions be guided by risk for future events as determined by medical history and estimated risk based on measured risk factors [3]. Claims-based algorithms to approximate clinical risk stratification may be useful in future studies of outcomes of treatment with novel LDL cholesterol lowering medications, in which comparison groups would need to be identified.

Schneeweiss and colleagues found that the ability of claims data to predict LDL cholesterol values was poor. They concluded that in settings where LDL cholesterol is a potential confounder, estimating missing LDL cholesterol values using claims may not substantially improve confounding control [10]. Similarly, we found that our algorithms did not identify representative groups of statin users at high risk for CHD events who had uncontrolled LDL cholesterol. The new guidelines have less focus on LDL treatment targets, but retain recommendations to monitor LDL treatment response [3]. This suggests that identifying comparison groups for pharmacovigilance studies that are comparable in their LDL cholesterol status and other characteristics may be difficult to accomplish using only claims data. It is also important in pharmacovigilance studies to identify comparison groups that would be similar in their risk for non-CHD adverse events. Further work is required in this area.

This study has limitations. First, our classifications of high risk groups were based partly on self-reported medical history in REGARDS. Participants over-reporting or under-reporting medical conditions may have resulted in misclassification of true high risk status, diluting the ability of algorithms to correctly identify target groups. Second, we did not incorporate Medicare Part D pharmacy claims into our algorithms. Part D was implemented in 2006, with high penetration in the Medicare population by 2007. However, most REGARDS in-home visits occurred before 2007, and few REGARDS participants had Part D coverage prior to their REGARDS in-home visit. Including Part D pharmacy claims may improve the ability to identify groups at high cardiovascular risk or with uncontrolled LDL cholesterol. Third, the sample of REGARDS participants linked to Medicare may not be representative of the overall Medicare population enrolled in Parts A and B, where we would hope to apply these algorithms. Also, Medicare Advantage plan enrollees were not included in our study, so our algorithms may not be generalizable to that subset of Medicare beneficiaries. Fourth, use of claims data for identifying health-related variables is limited by potential inaccuracies in the claims. For example, administrative coding of diagnoses and procedures may be affected by reimbursement incentives, random or systematic coding errors, or mismatches in diagnostic resolution of available codes versus diagnostic resolution in clinical practice; these potential biases may also fluctuate over time. This could be a particular problem for documenting behavioral risk factors like smoking. To maximize the accuracy of our algorithms we used previously validated claims-based definitions of diagnoses and procedures when such were available. Fifth, we used an area-level income variable from Census data as a pre-specified variable in our algorithms. Incorporating individual-level income data for Medicare beneficiaries could strengthen the algorithms.

## Conclusions

In summary, we have demonstrated that claims-based algorithms can be used to identify Medicare beneficiaries at high risk for CHD events. Despite not having clinical or laboratory data, Medicare claims have potential as a data source for pharmacovigilance studies when groups at high risk for CHD events need to be identified. Improving algorithms for identifying subsets of high risk or groups with uncontrolled LDL cholesterol will require further work. Representativeness and generalizability will need to be considered in interpreting the findings of future studies conducted in cohorts identified by these algorithms.

## Abbreviations

ATP III: National Cholesterol Education Program Adult Treatment Panel III; CHD: Coronary heart disease; HDL: High density lipoprotein; LDL: Low density lipoprotein; MI: Myocardial infarction; NPV: Negative predictive value; PPV: Positive predictive value; REGARDS: REasons for Geographic And Racial Differences in Stroke.

## Competing interests

This work was supported by Amgen, Inc. The authors conducted all analyses and maintained the rights to publish this manuscript. All authors have received research support from Amgen, Inc. In addition, JRC and PM have served as consultants for Amgen, Inc.

## Authors’ contributions

ELT participated in the conception and design of the study and the interpretation of the data and drafted the manuscript. PM participated in the conception and design of the study, the interpretation of the data, and revision of the manuscript for important intellectual content. HZ conducted the statistical analysis and participated in revision of the manuscript for important intellectual content. MMS participated in the collection of the data, interpretation of the data, and revision of the manuscript for important intellectual content. JRC participated in the collection of the data, interpretation of the data, and revision of the manuscript for important intellectual content. ED participated in interpretation of the data and revision of the manuscript for important intellectual content. VB participated in interpretation of the data and revision of the manuscript for important intellectual content. TMB participated in the collection of the data, interpretation of the data, and revision of the manuscript for important intellectual content. EBL participated in the conception and design of the study and the interpretation of the data and helped to draft the manuscript. All authors read and approved the final version.

## Pre-publication history

The pre-publication history for this paper can be accessed here:

http://www.biomedcentral.com/1472-6963/14/195/prepub

## Supplementary Material

Additional file 1 Supplemental Content. Click here for file
