# Supplementary material for: Claims-based algorithms for identifying Medicare beneficiaries at high estimated risk for coronary heart disease events: a cross-sectional study
Source: BMC Health Serv Res. 2014 Apr 29;14:195. doi: 10.1186/1472-6963-14-195 (PMC4101858; doi:10.1186/1472-6963-14-195)
Supplement: Additional file 1 — Supplemental Content. [file 1472-6963-14-195-S1.zip › Additional files/4404101981144583_add1.docx]

**SUPPLEMENTAL CONTENT**

**Claims-based algorithms for identifying Medicare beneficiaries at high estimated risk for coronary heart disease events: a cross-sectional study**

Evan L. Thacker, Paul Muntner, Hong Zhao, Monika M. Safford, Jeffrey R. Curtis, Elizabeth Delzell, Vera Bittner, Todd M. Brown, Emily B. Levitan

**TEXT: SUPPLEMENTAL METHODS**

REGARDS Measurements and Definitions 3

Pre-specified Medicare Variables 3

Data Mining Medicare Variables 6

References 7

**SUPPLEMENTAL FIGURES**

Supplemental Figure 1. Sensitivity, specificity, positive predictive value, and negative predictive value curves for Medicare claims-based algorithms for identifying high risk conditions defined in REGARDS study data 8

Supplemental Figure 2. Sensitivity, specificity, positive predictive value, and negative predictive value curves for Medicare claims-based algorithms for identifying uncontrolled LDL cholesterol defined in REGARDS study data among statin users at high risk for CHD events 9

Supplemental Figure 3. Sensitivity analyses: Distributions of predicted probabilities of having a high risk condition (estimated by Medicare data) by observed presence of absence of the high risk condition (REGARDS study data); and sensitivity, specificity, positive predictive value, and negative predictive value curves for Medicare claims-based algorithms for identifying high risk conditions defined in REGARDS study data 10

**SUPPLEMENTAL TABLES**

Supplemental Table 1. Participant characteristics from REGARDS data by observed (REGARDS) and model-predicted (Medicare) very high risk for CHD events, from the model with pre-specified Medicare variables only 11

Supplemental Table 2. Participant characteristics from REGARDS data by observed (REGARDS) and model-predicted (Medicare) Framingham 10-year CHD risk score >20%, from the model with pre-specified Medicare variables only 12

Supplemental Table 3. Participant characteristics from REGARDS data by observed (REGARDS) and model-predicted (Medicare) LDL cholesterol ≥100 mg/dL among statin users at high risk for CHD events, from the model with pre-specified Medicare variables only 13

Supplemental Table 4. Participant characteristics from REGARDS data by observed (REGARDS) and model-predicted (Medicare) LDL cholesterol ≥70 mg/dL among statin users at high risk for CHD events, from the model with pre-specified Medicare variables only 14

Supplemental Table 5. Claims-based models to identify high risk conditions and uncontrolled LDL cholesterol defined using REGARDS data, from models with pre-specified Medicare variables only 15

Supplemental Table 6. Sensitivity analyses: Test characteristics of Medicare claims-based models using modified definitions for identifying high risk groups defined in REGARDS study data 17

Supplemental Table 7. Sensitivity analyses: Test characteristics of Medicare claims-based models using one year of claims for identifying high risk groups defined in REGARDS study data 18

Supplemental Table 8. Sensitivity analyses: Test characteristics of Medicare claims-based models using one year of claims for identifying uncontrolled LDL cholesterol among statin users at high risk for CHD events defined in REGARDS study data 19

**SUPPLEMENTAL METHODS**

**REGARDS Measurements and Definitions**

The REGARDS study has been previously described.^1^ Age, sex, and race (black, white) were self-reported. Income was self-reported and categorized as <$20,000/y, $20,000-$34,999/y, $35,000-$74,999/y, ≥$75,000/y, or refused to answer. Education was self-reported and categorized as less than high school, high school graduate, some college, or college graduate or above. Participants’ home addresses were geocoded and geographic region was categorized as Northeast, Midwest, South, and West. Family history of myocardial infarction (MI) in the father before age 55 or in the mother before age 65 was self-reported. Cigarette smoking was self-reported and categorized as current, past, or never. Use of antihypertensive medication, oral hypoglycemic medication, and insulin was self-reported. Use of statins was determined during the REGARDS in-home visit by a medication inventory in which an examiner recorded all medications the participant had used in the prior 2 weeks based on pill bottle review.

Body weight, height, and waist circumference were measured and body mass index was calculated as weight[kg]/height[m^2^]. Systolic and diastolic blood pressure were the average of two measurements taken following a standardized protocol. Triglycerides, total cholesterol, HDL cholesterol, and glucose were measured by colorimeteric reflectance spectrophotometry, and C-reactive protein was measured by particle-enhanced immunonephelometry.^2^ LDL cholesterol was calculated with the Friedewald equation.^3^

Hypertension was defined as systolic blood pressure ≥140 mmHg, diastolic blood pressure ≥90 mmHg, or self-reported physician diagnosis of high blood pressure with use of antihypertensive medication. Metabolic syndrome was defined according to ATP III guidelines as having at least three of the following components: (a) waist circumference >102 cm (men) or >88 cm (women), (b) triglycerides ≥150 mg/dL, (c) HDL cholesterol <40 (men) or <50 (women), (d) systolic blood pressure ≥130 mmHg or diastolic blood pressure ≥85 or self-reported physician diagnosis of high blood pressure with use of antihypertensive medication, or (e) glucose ≥100 mg/dL or self-reported physician diagnosis of diabetes with use of oral hypoglycemic medication or insulin.^4^ Diabetes was defined as fasting glucose ≥126 mg/dL or self-reported physician diagnosis of diabetes with use of oral hypoglycemic medication or insulin.

History of CHD was defined as self-reported physician diagnosis of MI, evidence of MI on electrocardiogram, or self-reported coronary revascularization. Acute MI in the prior year was defined as self-reported physician diagnosis of MI that occurred less than one year before the telephone interview. Peripheral arterial disease, abdominal aortic aneurysm, and carotid artery disease were defined as self-reported surgeries or procedures to repair those arteries. Stroke was defined as self-reported physician diagnosis of stroke.

**Pre-specified Medicare Variables**

Using all Medicare data available prior to the REGARDS in-home visit, we defined the following pre-specified Medicare variables.

*Age:* Years of age at the time of the REGARDS in-home visit

*Sex:* Male or female (Social Security Administration or Railroad Retirement Board variable incorporated into Medicare data)

*Race:* Black, white, Hispanic, Asian, or other (Social Security Administration variable incorporated into Medicare data)

*Medicaid eligible:* State buy-In (value of ‘C’ from the entitlement variable) in all months for the year prior to the REGARDS in-home visit

*Area-level income:* Percent living below poverty in quintiles (2000 Census variable incorporated into Medicare data)

*Geographic region:* US Census region at the time of the REGARDS in-home visit – Northeast, Midwest, South, West

*Evidence of tobacco use:* At least 1 claim in any file type with ICD-9 diagnoses (any position) of 305.1 (tobacco use disorder) or V15.82 (history of tobacco use) or HCPCS codes G0375 or G0376 or CPT codes 99406 or 99407 (smoking cessation counseling)

*History of hyperlipidemia:* At least 2 claims in any file type on separate calendar days with ICD-9 diagnoses (any position) of 272.1, 272.2, or 272.4

*History of hypertension:* At least 2 claims in any file type on separate calendar days with ICD-9 diagnoses (any position) of 401.x

*History of diabetes:* Either one of the following:

1. At least 1 inpatient claim with discharge ICD-9 diagnoses (any position) of 250.xx, 357.2, 362.0x, or 366.41
2. At least 2 carrier claim, carrier line or outpatient claims with ICD-9 diagnoses (any position) of 250.xx, 357.2, 362.0x, or 366.41, linked by CLAIM_ID to an ambulatory physician evaluation and management claim, with the 2 claims occurring at least 7 days apart

*Acute MI^5^:* At least 1 inpatient claim with discharge ICD-9 diagnoses (first or second position) of 410.x0 or 410.x1

*Coronary revascularization:* At least 1 inpatient or outpatient claim or revenue center file or carrier line file with CPT codes 92980-92996 (angioplasty or stent) or 33510-33536 (CABG) or ICD-9 procedure codes 00.66 or 36.01-36.09 (angioplasty or stent) or 36.10-36.19 (CABG)

*History of CHD:* Any one of the following:

1. Acute MI as defined above
2. Coronary revascularization as defined above
3. Other ischemic heart disease: Either one of the following:
4. At least 1 inpatient claim with ICD-9 diagnoses (any position) of 411.xx, 412.00, 413.xx, or 414.xx
5. At least 2 carrier claim, carrier line or outpatient claims with ICD-9 diagnoses (any position) of 411.xx, 412.00, 413.xx, or 414.xx, linked by CLAIM_ID to an ambulatory physician evaluation and management claim, with the 2 claims occurring at least 7 days apart

*History of stroke:* Any one of the following:

1. At least 1 inpatient ICD-9 diagnosis (any position) of 430.xx, 431.xx, 433.x1, 434.x1 or 436.x
2. At least 1 outpatient, carrier claim, or carrier line with ICD-9 diagnoses (any position) of 430.xx, 431.xx, 433.x1, 434.x1 or 436.x, linked by CLAIM_ID to an ambulatory physician evaluation and management claim

*History of abdominal aortic aneurism^6^:* Either one of the following:

1. At least 1 inpatient claim with ICD-9 diagnoses (any position) of 441.3-441.9 or CPT codes 34800-34834 or ICD-9 procedure codes 38.44, 39.25, or 39.71
2. At least 2 carrier claim, carrier line or outpatient claims on separate calendar days with ICD-9 diagnoses (any position) of 441.3-441.9 or ICD-9 procedure codes 38.44, 39.25, or 39.71

*History of peripheral arterial disease^7,8^:* Any one of the following:

1. At least 1 inpatient claim with ICD-9 diagnoses (primary diagnosis) of 440.20-440.24, 440.31, 444.2, 443.9, or 444.81
2. At least 2 carrier claim, carrier line or outpatient claims on separate calendar days with ICD-9 diagnoses (primary diagnosis) of 440.20-440.24, 440.31, 444.2, 443.9, 444.2, or 444.81
3. At least 1 claim in any file type with CPT code 37205 or 75962

*History of carotid artery disease^9^:* Either one of the following:

1. At least 1 inpatient claim with ICD-9 diagnoses (primary diagnosis) of 433.10, 433.11, 433.30, 433.31, or CPT code 35301, 37215, 37216, or ICD-9 procedure code 00.61 or 00.63
2. At least 2 carrier claim, carrier line or outpatient claims on separate calendar days with ICD-9 diagnoses (primary diagnosis) 433.10, 433.11, 433.30, 433.31, or CPT code 35301, 37215, 37216, or ICD-9 procedure code 00.61 or 00.63

*Cardiologist care:* At least 1 claim with provider specialty code 06 (Physician / Cardiovascular Disease [Cardiology])

*Endocrinologist care:* At least 1 claim with provider specialty code 46 (Physician / Endocrinology)

*Neurologist care:* At least 1 claim with provider specialty code 13 (Physician / Neurology)

*Number of evaluation and management visits:* Number of different calendar days with claims with ambulatory (outpatient or emergency department) HCPCS codes for evaluation and management

*Hospitalization for any cause:* At least 1 inpatient claim or at least 1 inpatient physician evaluation and management code

*Cardiac stress test^10^:* At least 1 claim in any file type with CPT codes 93015, 93016, 93017, 93018, 93350, 93351, 78452, 78465 or ICD-9 procedure codes 89.41-89.44 (includes stress echocardiogram)

*Echocardiogram^11^:* At least 1 claim in any file type with CPT codes 93306, 93307, 93320, or 93325, or ICD-9 procedure code 88.72, or HCPCS codes C8923, C8924, C8928, C8929, or C8930

*Electrocardiogram^12,13^:* At least 1 claim in any file type with CPT codes 93000, 93005, 93010, 93040, 93041, 93042, 93224, 93225, 93226, or 93227, or ICD-9 procedure codes 89.50, 89.51, or 89.52 (includes Holter monitoring)

**Data Mining Medicare Variables**

For each of the five conditions we sought to identify using Medicare variables, we used the following data mining procedure, adapted from a previously described algorithm,^14^ to identify additional Medicare variables beyond those we had pre-specified. The data mining procedure is described in Steps 1-4 below for the Condition 1, high risk for CHD events.

1. Identify Medicare codes from four dimensions: (i) inpatient diagnosis codes, (ii) outpatient, carrier line, and carrier claim diagnosis codes, (iii) inpatient and outpatient procedure codes, (iv) outpatient and carrier line HCPSC codes.
2. Calculate the prevalence of each code. Subtract prevalences >50% from 100% to obtain symmetric prevalence. Drop codes observed in fewer than 50 participants. Select the 50 most prevalent codes in each dimension (200 total variables). For each selected code determine whether each participant received the code more than once, more than the median number of times, or more than the 75^th^ percentile (600 total variables).
3. Using logistic regression models with high risk as the dependent variable and each data mining Medicare variable included separately as the independent variable, obtain the unadjusted odds ratio of high risk for each data mining Medicare variable. Take the inverse of odds ratios <1 to obtain symmetric odds ratios.
4. Rank all 600 data mining variables by the product of the symmetric prevalence from Step 2 and the natural logarithm of the symmetric odds ratio from Step 3. To avoid over-fitting, select a number of variables equal to 5% of the sample size with high risk = 1 or 5% of the sample size with high risk = 0, whichever is smaller. Include the selected data mining variables as independent variables in the expanded model for identifying high risk. Remove data mining variables with a variance inflation factor ≥10 to reduce collinearity.

**References**

1. Howard VJ, Cushman M, Pulley L, et al. The Reasons for Geographic and Racial Differences in Stroke study: objectives and design. Neuroepidemiology. 2005;25:135-143.
2. Cushman M, McClure LA, Howard VJ, Jenny NS, Lakoski SG, Howard G. Implications of increased C-reactive protein for caridiovascular risk stratification in black and white men and women in the US. Clin Chem. 2009;55:1627-1636.
3. Friedewald WT, Levy RI, Fredrickson DS. Estimation of the concentration of low-density lipoprotein cholesterol in plasma, without use of the preparative ultracentrifuge. Clin Chem. 1972;6:499-502.
4. National Cholesterol Education Program. Third Report of the National Cholesterol Education Program (NCEP) Expert Panel on Detection, Evaluation, and Treatment of High Blood Cholesterol in Adults (Adult Treatment Panel III) Final Report. URL: http://www.nhlbi.nih.gov/guidelines/cholesterol/atp3_rpt.htm. Accessed 9 Aug 2012.
5. Cutrona SL, Toh S, Iyer A, et al. Design for validation of acute myocardial infarction cases in Mini-Sentinel. Pharmacoepidemiol Drug Saf. 2012;21:274-281.
6. Trends and Regional Variations in Abdominal Aortic Aneurysm Repair. 2006. URL: http://www.dartmouthatlas.org/downloads/reports/AAA_report_2006.pdf. Accessed 13 Jun 2012.
7. Harris TJ, Zafar AM, Murphy TP. Utilization of Lower Extremity Arterial Disease Diagnostic and Revascularization Procedures in Medicare Beneficiaries 2000–2007. AJR Am J Roentgenol. 2011;197:W314-W317.
8. Hirsch AT, Hartman L, Town RJ, Virnig BA. National healthcare costs of peripheral arterial disease in the Medicare population. Vasc Med. 2008;13:209-215.
9. Carotid artery stenting procedures: reimbursement information. 2008. URL: http://www.bostonscientific.com/templatedata/imports/collateral/Reimbursement/Peripheral_Interventions/rmbgde_CAS_ProcGuide_01_us.pdf. Accessed 18 Jun 2012.
10. CPT codes and reimbursement: cardiac stress testing. 2011. URL: http://www.midmark.com/Marketing%20Collateral/CPT-Stress.pdf. Accessed 18 Jun 2012.
11. Billing and Coding Guidelines for Transthoracic Echocardiography TTE (CV-026). 2009. URL: http://downloads.cms.gov/medicare-coverage-database/lcd_attachments/28565_28/l28565_cv026_cbg_10012010.pdf. Accessed 18 Jun 2012.
12. ECG reimbursement. 2010. URL: http://www.qrssys.com/194050.ihtml. Accessed 18 Jun 2012.
13. CPT coding options for Holter monitoring. 2011. URL: http://www.advancedbiosensor.com/downloads/CPT%20Coding%20Options%20for%20Holter%20Monitoring.pdf. Accessed 18 Jun 2012.
14. Schneeweiss S, Rassen JA, Glynn RJ, Avorn J, Mogun H, Brookhart MA. High-dimensional propensity score adjustment in studies of treatment effects using healthcare claims data. Epidemiology. 2009;20:512-522.

**SUPPLEMENTAL FIGURES**

**Supplemental Figure 1. Sensitivity, specificity, positive predictive value, and negative predictive value curves for Medicare claims-based algorithms for identifying high risk conditions defined in REGARDS study data**


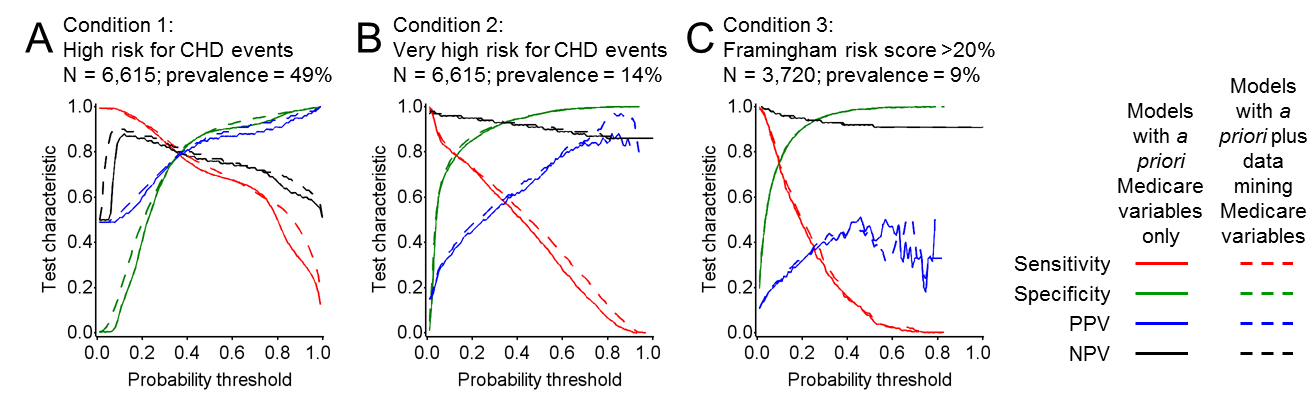


Panel A shows Condition 1, high risk for CHD events, among all eligible participants. Panel B shows Condition 2, very high risk for CHD events, among all eligible participants. Panel C shows Condition 3, Framingham CHD risk score >20%, among eligible participants without a history of CHD or risk equivalents. In each panel the sold curves represent models using pre-specified Medicare variables only, and the dashed curves represent models using pre-specified plus data mining Medicare variables. Red curves represent sensitivity, green curves represent specificity, blue curves represent positive predictive value, and black curves represent negative predictive value. Probability threshold, ranging from 0 to 1, refers to dichotomizing the predicted probabilities at different thresholds to calculate the test characteristics of sensitivity, specificity, positive predictive value, and negative predictive value. The test characteristics change depending on the predicted probability threshold chosen. For example, see Table 2 in the main text for the probability thresholds that yield 90% uncorrected specificity.

**Supplemental Figure 2. Sensitivity, specificity, positive predictive value, and negative predictive value curves for Medicare claims-based algorithms for identifying uncontrolled LDL cholesterol defined in REGARDS study data among statin users at high risk for CHD events**


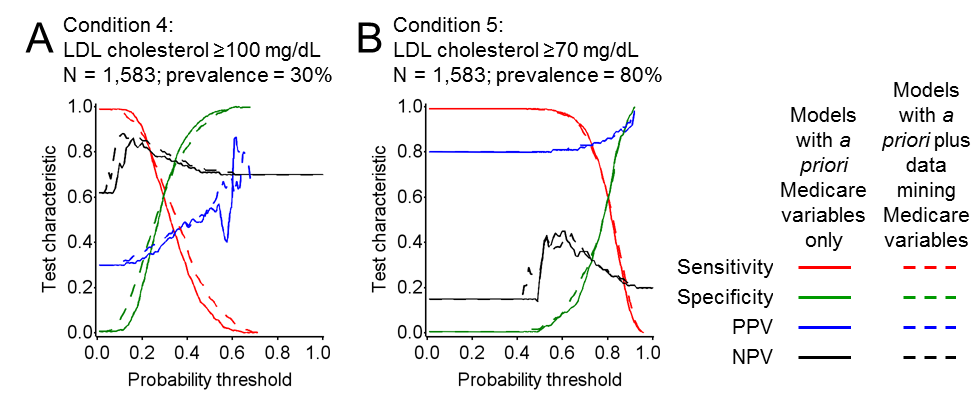


Panel A shows Condition 4, LDL cholesterol ≥100 mg/dL, among eligible participants at high risk for CHD events who were using statins according to the REGARDS in-home visit medication inventory. Panel B shows Condition 5, LDL cholesterol ≥70 mg/dL, among eligible participants at high risk for CHD events who were using statins according to the REGARDS in-home visit medication inventory. In each panel the sold curves represent models using pre-specified Medicare variables only, and the dashed curves represent models using pre-specified plus data mining Medicare variables. Red curves represent sensitivity, green curves represent specificity, blue curves represent positive predictive value, and black curves represent negative predictive value. Probability threshold, ranging from 0 to 1, refers to dichotomizing the predicted probabilities at different thresholds to calculate the test characteristics of sensitivity, specificity, positive predictive value, and negative predictive value. The test characteristics change depending on the predicted probability threshold chosen. For example, see Table 2 in the main text for the probability thresholds that yield 90% uncorrected specificity.

**Supplemental Figure 3. Sensitivity analyses: Distributions of predicted probabilities of having a high risk condition (estimated by Medicare data) by observed presence of absence of the high risk condition (REGARDS study data); and sensitivity, specificity, positive predictive value, and negative predictive value curves for Medicare claims-based algorithms for identifying high risk conditions defined in REGARDS study data**


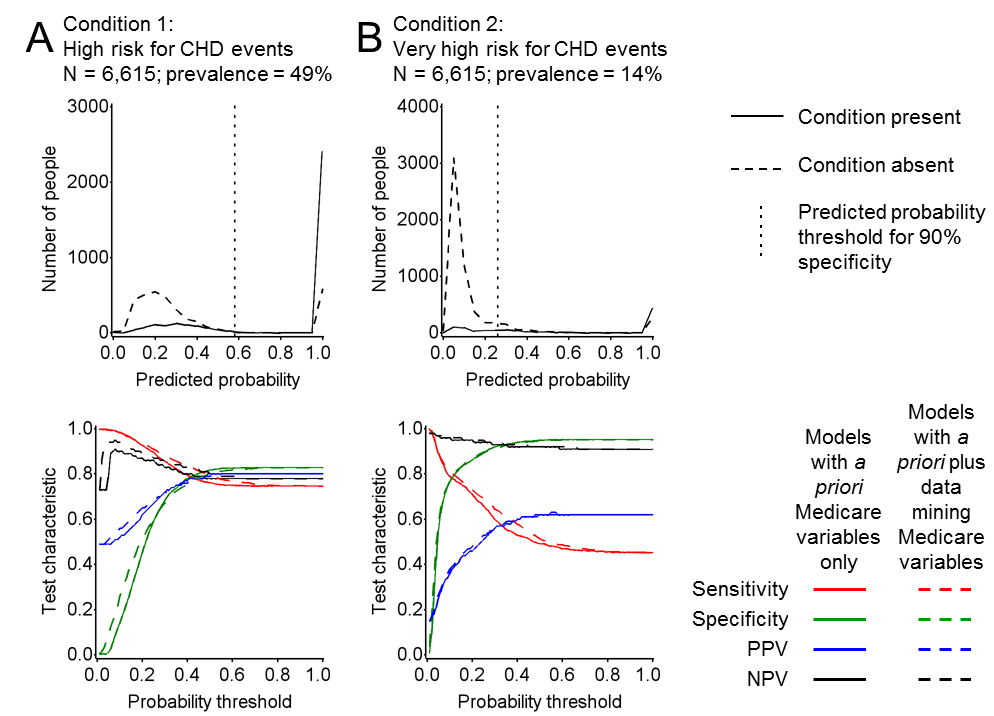


Panel A shows Condition 1, high risk for CHD events. We assigned a predicted probability of 1 for participants who met a pre-specified claims-based definition of high risk for CHD events, and model-based predicted probabilities otherwise. Panel B shows Condition 2, very high risk for CHD events. We assigned a predicted probability of 1 for participants who met a pre-specified claims-based definition of very high risk for CHD events, and model-based predicted probabilities otherwise.

**SUPPLEMENTAL TABLES**

**Supplemental Table 1. Participant characteristics from REGARDS data by observed (REGARDS) and model-predicted (Medicare) very high risk for CHD events, from the model with pre-specified Medicare variables only**

|  |  | **Model-predicted very high risk**  **(Medicare)** | | **Model-predicted not very high risk**  **(Medicare)** | |
| --- | --- | --- | --- | --- | --- |
| **Characteristic*** | **Overall**  **N = 6,615** | **Observed very high risk (REGARDS)**  **(true positives)**  **N = 613** | **Observed not very high risk (REGARDS)**  **(false positives)**  **N = 563** | **Observed very high risk (REGARDS)**  **(false negatives)**  **N = 340** | **Observed not very high risk (REGARDS)**  **(true negatives)**  **N = 5,099** |
| Age, y | 73.2 (5.6) | 73.2 (5.3) | 74.1 (5.3) | 73.6 (5.7) | 73.1 (5.6) |
| Male | 49.6 | 67.4 | 71.2 | 53.2 | 44.8 |
| Black | 30.4 | 27.1 | 26.6 | 32.1 | 31.1 |
| Income <$35,000/y | 56.7 | 59.7 | 55.4 | 61.6 | 56.1 |
| Education ≤ High school graduate | 40.6 | 46.3 | 42.9 | 48.4 | 39.2 |
| Current use of statins | 37.6 | 69.2 | 60.9 | 46.2 | 30.7 |
| Total cholesterol, mg/dL | 187.0 (38.9) | 166.8 (35.8) | 171.1 (40.7) | 182.8 (38.1) | 191.4 (37.9) |
| LDL cholesterol, mg/dL | 109.8 (34.0) | 93.5 (31.3) | 99.2 (34.3) | 107.1 (32.3) | 113.1 (33.6) |
| LDL cholesterol among statin non-users, mg/dL | 120.4 (33.9) | 109.5 (37.0) | 116.9 (39.0) | 119.6 (32.2) | 121.2 (33.4) |
| HDL cholesterol, mg/dL | 52.1 (16.4) | 43.5 (12.9) | 49.2 (14.8) | 46.3 (14.3) | 53.8 (16.6) |
| Triglycerides, mg/dL | 125.3 (61.5) | 149.3 (71.8) | 113.9 (53.3) | 147.5 (70.9) | 122.2 (59.3) |
| Family history of MI | 19.0 | 26.1 | 21.2 | 25.1 | 17.6 |
| Current cigarette smoking | 8.9 | 13.7 | 5.0 | 23.8 | 7.8 |
| Body mass index, kg/m^2^ | 28.1 (5.4) | 29.9 (5.2) | 27.9 (5.1) | 29.3 (5.6) | 27.9 (5.4) |
| Systolic blood pressure, mm Hg | 130.3 (16.6) | 132.5 (18.0) | 130.7 (18.0) | 133.1 (17.2) | 129.7 (16.2) |
| Diastolic blood pressure, mm Hg | 75.3 (9.4) | 74.0 (10.1) | 74.6 (9.4) | 75.9 (9.7) | 75.5 (9.2) |
| Blood glucose, mg/dL | 101.4 (27.9) | 118.9 (45.1) | 100.6 (25.4) | 108.8 (28.9) | 98.9 (24.4) |
| C-reactive protein, mg/L | 4.5 (8.5) | 5.5 (11.3) | 4.5 (8.6) | 6.4 (11.1) | 4.2 (7.9) |
| Hypertension | 64.7 | 79.3 | 68.0 | 79.1 | 61.7 |
| Metabolic syndrome | 39.0 | 77.8 | 24.7 | 77.1 | 33.4 |
| Diabetes | 20.8 | 57.6 | 22.0 | 30.9 | 15.6 |
| Coronary heart disease | 24.6 | 100.0 | 54.4 | 100.0 | 7.2 |
| Acute MI in prior year | 1.3 | 13.9 | 0.0† | 3.8 | 0.0† |
| Peripheral arterial disease | 2.6 | 8.4 | 4.1 | 4.1 | 1.6 |
| Abdominal aortic aneurysm | 1.6 | 3.9 | 6.0 | ‡ | ‡ |
| Carotid artery disease | 3.2 | 11.7 | 7.6 | 5.0 | 1.6 |
| Stroke | 7.6 | 14.4 | 11.2 | 12.6 | 6.1 |

Abbreviations: HDL, high density lipoprotein; LDL, low density lipoprotein; MI, myocardial infarction.

* Numbers are column percentages or means (standard deviations). Income was missing for 914 participants, education for 4, body mass index for 16, and C-reactive protein for 164.

† By definition, participants not at very high risk for CHD events did not have acute MI in the prior year according to REGARDS study data.

‡ The Centers for Medicare and Medicaid Services (CMS) requires the figure be redacted because the cell contained fewer than 11 participants, or would allow a number fewer than 11 participants to be deduced in another cell.

**Supplemental Table 2. Participant characteristics from REGARDS data by observed (REGARDS) and model-predicted (Medicare) Framingham 10-year CHD risk score >20%, from the model with pre-specified Medicare variables only**

|  |  | **Model-predicted risk score >20%**  **(Medicare)** | | **Model-predicted risk score ≤20%**  **(Medicare)** | |
| --- | --- | --- | --- | --- | --- |
| **Characteristic*** | **Overall**  **N = 3,720** | **Observed risk score >20% (REGARDS)**  **(true positives)**  **N = 163** | **Observed risk score ≤20% (REGARDS)**  **(false positives)**  **N = 345** | **Observed risk score >20% (REGARDS)**  **(false negatives)**  **N = 166** | **Observed risk score ≤20% (REGARDS)**  **(true negatives)**  **N = 3,046** |
| Age, y | 72.9 (5.5) | 77.7 (5.1) | 77.6 (6.0) | 74.4 (5.3) | 72.1 (5.1) |
| Male | 43.3 | ‡ | 95.9 | 63.3 | ‡ |
| Black | 27.6 | 19.6 | 22.9 | 27.7 | 28.6 |
| Income <$35,000/y | 54.6 | 49.3 | 45.9 | 61.2 | 55.6 |
| Education ≤ High school graduate | 36.7 | 38.7 | 33.5 | 45.8 | 36.4 |
| Current use of statins | 25.9 | 17.8 | 23.2 | 18.1 | 27.1 |
| Total cholesterol, mg/dL | 195.0 (37.5) | 179.7 (36.9) | 182.5 (33.4) | 201.8 (40.9) | 196.9 (37.3) |
| LDL cholesterol, mg/dL | 116.2 (33.2) | 113.7 (31.9) | 108.8 (29.7) | 129.6 (36.0) | 116.5 (33.3) |
| LDL cholesterol among statin non-users, mg/dL | 122.6 (32.8) | 118.7 (32.2) | 113.3 (30.1) | 133.7 (35.7) | 123.3 (32.7) |
| HDL cholesterol, mg/dL | 54.8 (16.7) | 37.4 (6.6) | 51.0 (15.8) | 40.3 (9.9) | 56.9 (16.5) |
| Triglycerides, mg/dL | 120.1 (57.6) | 142.9 (61.1) | 113.6 (58.8) | 159.3 (70.1) | 117.5 (55.5) |
| Family history of MI | 17.2 | 19.0 | 9.2 | 11.1 | 18.4 |
| Current cigarette smoking | 7.6 | 11.0 | ‡ | 17.5 | ‡ |
| Body mass index, kg/m^2^ | 27.5 (5.2) | 27.2 (4.1) | 26.6 (4.0) | 28.9 (4.5) | 27.5 (5.4) |
| Systolic blood pressure, mm Hg | 129.0 (15.9) | 141.6 (15.2) | 128.6 (14.0) | 147.7 (15.8) | 127.3 (15.2) |
| Diastolic blood pressure, mm Hg | 75.5 (9.1) | 79.0 (11.4) | 74.9 (8.5) | 80.5 (9.2) | 75.1 (9.0) |
| Blood glucose, mg/dL | 93.0 (10.6) | 95.4 (12.1) | 93.0 (11.3) | 97.0 (11.3) | 92.7 (10.3) |
| C-reactive protein, mg/L | 4.0 (7.5) | 4.1 (6.3) | 3.9 (11.3) | 4.9 (7.7) | 4.0 (7.0) |
| Hypertension | 57.8 | 90.2 | 64.1 | 86.7 | 53.8 |
| Metabolic syndrome | 27.8 | 52.1 | 20.0 | 70.5 | 25.1 |
| Diabetes | 0.0† | 0.0† | 0.0† | 0.0† | 0.0† |
| Coronary heart disease | 0.0† | 0.0† | 0.0† | 0.0† | 0.0† |
| Acute MI in prior year | 0.0† | 0.0† | 0.0† | 0.0† | 0.0† |
| Peripheral arterial disease | 0.0† | 0.0† | 0.0† | 0.0† | 0.0† |
| Abdominal aortic aneurysm | 0.0† | 0.0† | 0.0† | 0.0† | 0.0† |
| Carotid artery disease | 0.0† | 0.0† | 0.0† | 0.0† | 0.0† |
| Stroke | 0.0† | 0.0† | 0.0† | 0.0† | 0.0† |

Abbreviations: HDL, high density lipoprotein; LDL, low density lipoprotein; MI, myocardial infarction.

* Numbers are column percentages or means (standard deviations). Income was missing for 528 participants, education for 1, body mass index for 6, and C-reactive protein for 88.

† By definition, participants in this analysis did not have a history of CHD or risk equivalents according to REGARDS study data.

‡ The Centers for Medicare and Medicaid Services (CMS) requires the figure be redacted because the cell contained fewer than 11 participants, or would allow a number fewer than 11 participants to be deduced in another cell.

**Supplemental Table 3. Participant characteristics from REGARDS data by observed (REGARDS) and model-predicted (Medicare) LDL cholesterol ≥100 mg/dL among statin users at high risk for CHD events, from the model with pre-specified Medicare variables only**

|  |  | **Model-predicted LDL ≥100 mg/dL**  **(Medicare)** | | **Model-predicted LDL <100 mg/dL**  **(Medicare)** | |
| --- | --- | --- | --- | --- | --- |
| **Characteristic*** | **Overall**  **N = 1,583** | **Observed LDL ≥100 mg/dL (REGARDS)**  **(true positives)**  **N = 105** | **Observed LDL <100 mg/dL (REGARDS)**  **(false positives)**  **N = 108** | **Observed LDL ≥100 mg/dL (REGARDS)**  **(false negatives)**  **N = 374** | **Observed LDL <100 mg/dL (REGARDS)**  **(true negatives)**  **N = 996** |
| Age, y | 73.6 (5.4) | 72.1 (4.7) | 71.6 (4.9) | 73.5 (5.4) | 73.9 (5.5) |
| Male | 62.9 | 34.3 | 33.3 | 62.3 | 69.4 |
| Black | 29.5 | 91.4 | 94.4 | 25.9 | 17.3 |
| Income <$35,000/y | 54.0 | 72.7 | 77.8 | 53.6 | 49.8 |
| Education ≤ High school graduate | 41.7 | 57.2 | 57.4 | 42.5 | 38 |
| Current use of statins | 100.0† | 100.0† | 100.0† | 100.0† | 100.0† |
| Total cholesterol, mg/dL | 162.6 (30.0) | 195.6 (26.2) | 151.4 (20.0) | 192.4 (24.2) | 149.2 (21.4) |
| LDL cholesterol, mg/dL | 89.2 (24.9) | 121.3 (21.6) | 78.1 (13.7) | 117.2 (17.8) | 76.5 (14.9) |
| HDL cholesterol, mg/dL | 47.5 (14.0) | 51.4 (13.6) | 51.3 (15.9) | 46.7 (12.1) | 47.0 (14.3) |
| Triglycerides, mg/dL | 129.5 (63.3) | 114.6 (46.8) | 109.7 (54.7) | 142.7 (64.4) | 128.3 (64.2) |
| Family history of MI | 22.4 | 14.3 | 23.6 | 27.6 | 21.1 |
| Current cigarette smoking | 9.2 | 8.6 | 14.8 | 9.1 | 8.7 |
| Body mass index, kg/m^2^ | 29.1 (5.3) | 30.4 (5.6) | 30.6 (5.9) | 29.1 (5.1) | 28.8 (5.2) |
| Systolic blood pressure, mm Hg | 131.5 (17.7) | 132.4 (17.5) | 134.7 (17.9) | 133.3 (18.8) | 130.4 (17.2) |
| Diastolic blood pressure, mm Hg | 74.4 (9.8) | 76.0 (9.3) | 76.0 (12.0) | 76.2 (10.0) | 73.4 (9.3) |
| Blood glucose, mg/dL | 110.1 (36.3) | 111.7 (36.3) | 112 (40.1) | 110 (36.4) | 109.8 (35.8) |
| C-reactive protein, mg/L | 4.2 (8.4) | 4.4 (4.9) | 4.9 (8.5) | 4.8 (10.3) | 3.9 (7.9) |
| Hypertension | 76.6 | 81.0 | 88.9 | 76.5 | 74.9 |
| Metabolic syndrome | 53.7 | 55.2 | 63.0 | 58.3 | 50.8 |
| Diabetes | 44.4 | 49.5 | 47.2 | 42.5 | 44.3 |
| Coronary heart disease | 62.6 | 54.3 | 51.9 | 61.0 | 65.3 |
| Acute MI in prior year | 4.2 | ‡ | ‡ | 4.1 | 4.5 |
| Peripheral arterial disease | 5.9 | ‡ | ‡ | 5.9 | 5.6 |
| Abdominal aortic aneurysm | 4.3 | ‡ | ‡ | 4.0 | 4.9 |
| Carotid artery disease | 8.9 | 11.4 | ‡ | 11.2 | ‡ |
| Stroke | 15.7 | 18.1 | 18.5 | 17.1 | 14.6 |

Abbreviations: HDL, high density lipoprotein; LDL, low density lipoprotein; MI, myocardial infarction.

* Numbers are column percentages or means (standard deviations). Income was missing for 197 participants, education for 1, body mass index for 5, and C-reactive protein for 31.

† By definition, all participants in this analysis were current statin users according to REGARDS study data.

‡ The Centers for Medicare and Medicaid Services (CMS) requires the figure be redacted because the cell contained fewer than 11 participants, or would allow a number fewer than 11 participants to be deduced in another cell.

**Supplemental Table 4. Participant characteristics from REGARDS data by observed (REGARDS) and model-predicted (Medicare) LDL cholesterol ≥70 mg/dL among statin users at high risk for CHD events, from the model with pre-specified Medicare variables only**

|  |  | **Model-predicted LDL ≥70 mg/dL**  **(Medicare)** | | **Model-predicted LDL <70 mg/dL**  **(Medicare)** | |
| --- | --- | --- | --- | --- | --- |
| **Characteristic*** | **Overall**  **N = 1,583** | **Observed LDL ≥70 mg/dL (REGARDS)**  **(true positives)**  **N = 258** | **Observed LDL <70 mg/dL (REGARDS)**  **(false positives)**  **N = 33** | **Observed LDL ≥70 mg/dL (REGARDS)**  **(false negatives)**  **N = 1010** | **Observed LDL <70 mg/dL (REGARDS)**  **(true negatives)**  **N = 282** |
| Age, y | 73.6 (5.4) | 72.9 (5.5) | 72.7 (4.7) | 73.6 (5.4) | 73.9 (5.5) |
| Male | 62.9 | 68.2 | ‡ | 61.7 | ‡ |
| Black | 29.5 | 61.2 | 42.4 | 23.3 | 21.3 |
| Income <$35,000/y | 54.0 | 63.8 | 55.2 | 53.0 | 48.4 |
| Education ≤ High school graduate | 41.7 | 47.5 | 54.5 | 41.6 | 34.8 |
| Current use of statins | 100.0† | 100.0† | 100.0† | 100.0† | 100.0† |
| Total cholesterol, mg/dL | 162.6 (30.0) | 172.8 (27.6) | 135.4 (20.2) | 169.6 (27.0) | 131.3 (19.6) |
| LDL cholesterol, mg/dL | 89.2 (24.9) | 101.1 (23.1) | 57.2 (11.9) | 95.8 (20.7) | 58.2 (9.5) |
| HDL cholesterol, mg/dL | 47.5 (14.0) | 47.7 (13.9) | 51.2 (15.9) | 47.2 (13.2) | 48.2 (16.3) |
| Triglycerides, mg/dL | 129.5 (63.3) | 120.1 (57.7) | 135.2 (83.7) | 133.2 (62.1) | 124.3 (68.2) |
| Family history of MI | 22.4 | 22.3 | ‡ | 22.7 | ‡ |
| Current cigarette smoking | 9.2 | 13.6 | ‡ | 8.6 | ‡ |
| Body mass index, kg/m^2^ | 29.1 (5.3) | 28.9 (5.0) | 29.2 (6.0) | 29.2 (5.4) | 28.8 (4.7) |
| Systolic blood pressure, mm Hg | 131.5 (17.7) | 133.5 (18.6) | 131.9 (15.0) | 131.6 (17.8) | 129.3 (16.4) |
| Diastolic blood pressure, mm Hg | 74.4 (9.8) | 76.8 (10.6) | 74.6 (9.7) | 74.4 (9.6) | 72.3 (9.0) |
| Blood glucose, mg/dL | 110.1 (36.3) | 105.5 (28.5) | 116.5 (31.0) | 110.1 (37.9) | 113.5 (36.9) |
| C-reactive protein, mg/L | 4.2 (8.4) | 5.3 (12.0) | 2.6 (3.5) | 4.0 (7.6) | 4.0 (7.8) |
| Hypertension | 76.6 | 79.5 | ‡ | 75.7 | ‡ |
| Metabolic syndrome | 53.7 | 46.1 | 57.6 | 55.3 | 54.3 |
| Diabetes | 44.4 | 34.1 | 54.5 | 45.1 | 50.0 |
| Coronary heart disease | 62.6 | 46.9 | 45.5 | 64.4 | 72.7 |
| Acute MI in prior year | 4.2 | ‡ | ‡ | 4.9 | 4.7 |
| Peripheral arterial disease | 5.9 | 7.0 | ‡ | 6.3 | ‡ |
| Abdominal aortic aneurysm | 4.3 | 2.7 | ‡ | 4.6 | ‡ |
| Carotid artery disease | 8.9 | 11.2 | ‡ | 8.8 | ‡ |
| Stroke | 15.7 | 18.2 | ‡ | 15.0 | ‡ |

Abbreviations: HDL, high density lipoprotein; LDL, low density lipoprotein; MI, myocardial infarction.

* Numbers are column percentages or means (standard deviations). Income was missing for 197 participants, education for 1, body mass index for 5, and C-reactive protein for 31.

† By definition, all participants in this analysis were current statin users according to REGARDS study data.

‡ The Centers for Medicare and Medicaid Services (CMS) requires the figure be redacted because the cell contained fewer than 11 participants, or would allow a number fewer than 11 participants to be deduced in another cell.

**Supplemental Table 5. Claims-based models to identify high risk conditions and uncontrolled LDL cholesterol defined using REGARDS data, from models with pre-specified Medicare variables only**

|  | **Condition defined using REGARDS data** | | | | |
| --- | --- | --- | --- | --- | --- |
| **Pre-specified Medicare variables*** | **Condition 1:**  **High risk for CHD events**  **(N = 6,615)** | **Condition 2:**  **Very high risk for CHD events**  **(N = 6,615)** | **Condition 3:**  **Framingham CHD risk score >20%**  **(N = 3,720)** | **Condition 4:**  **LDL cholesterol ≥100 mg/dL**  **(N = 1,583)** | **Condition 5:**  **LDL cholesterol ≥70 mg/dL**  **(N = 1,583)** |
| Intercept | -2.52 (0.48) | -1.36 (0.64) | -10.15 (0.91) | 1.10 (0.96) | 3.56 (1.04) |
| Age, y | 0.02 (0.01) | -0.03 (0.01) | 0.11 (0.01) | -0.01 (0.01) | 0.00 (0.01) |
| Female vs. Male | -1.13 (0.10) | -0.39 (0.11) | -2.43 (0.23) | -0.08 (0.24) | -0.18 (0.15) |
| Race |  |  |  |  |  |
| White vs. Black | 0.12 (0.08) | 0.35 (0.11) | 0.32 (0.16) | -0.70 (0.14) | -0.48 (0.17) |
| Other vs. Black | 1.02 (0.63) | 0.48 (0.73) | 2.16 (0.96) | 0.04 (0.95) | -0.02 (1.15) |
| Medicaid eligible | 0.26 (0.14) | -0.03 (0.17) | -0.16 (0.41) | 0.12 (0.22) | -0.41 (0.25) |
| Area-level income† |  |  |  |  |  |
| Quintile 2 vs. 1 | -0.05 (0.11) | 0.05 (0.14) | -0.31 (0.21) | -0.08 (0.18) | 0.02 (0.23) |
| Quintile 3 vs. 1 | -0.05 (0.11) | -0.03 (0.14) | -0.49 (0.22) | -0.06 (0.19) | -0.19 (0.23) |
| Quintile 4 vs. 1 | -0.21 (0.11) | -0.16 (0.14) | -0.62 (0.22) | -0.01 (0.19) | -0.35 (0.23) |
| Quintile 5 vs. 1 | -0.21 (0.11) | -0.11 (0.15) | -0.42 (0.22) | -0.24 (0.20) | -0.51 (0.23) |
| Geographic region |  |  |  |  |  |
| Midwest vs. Northeast | 0.15 (0.16) | 0.03 (0.22) | 0.48 (0.29) | 0.05 (0.28) | -0.43 (0.37) |
| South vs. Northeast | -0.07 (0.15) | 0.09 (0.20) | -0.19 (0.27) | -0.22 (0.26) | -0.56 (0.33) |
| West vs. Northeast | -0.21 (0.19) | 0.08 (0.26) | -0.28 (0.36) | -0.34 (0.35) | -0.51 (0.41) |
| Evidence of tobacco use | -0.56 (0.33) | 0.36 (0.11) | -0.19 (0.24) | -0.08 (0.15) | -0.04 (0.17) |
| Hyperlipidemia | -0.70 (0.22) | -0.09 (0.09) | -1.42 (0.41) | -0.59 (0.39) | 0.03 (0.16) |
| Hypertension | 0.73 (0.08) | 0.56 (0.13) | 1.38 (0.16) | -0.14 (0.18) | -0.07 (0.21) |
| Diabetes | 1.55 (0.30) | 1.33 (0.10) | -0.40 (0.29) | -0.32 (0.13) | -0.56 (0.14) |
| Acute myocardial infarction | 0.84 (0.49) | 0.47 (0.19) | -12.41 (1484.90) | -0.09 (0.25) | -0.03 (0.25) |
| Coronary revascularization | -10.18 (240.70) | 0.58 (0.15) | -14.52 (1786.90) | -0.31 (0.18) | -0.46 (0.18) |
| Coronary heart disease | 3.23 (0.31) | 2.10 (0.11) | 0.02 (0.24) | 0.13 (0.15) | -0.35 (0.18) |
| Stroke | 1.37 (0.15) | -0.01 (0.15) | -0.63 (0.48) | -0.21 (0.18) | -0.27 (0.21) |
| Abdominal aortic aneurysm | 0.79 (0.27) | -0.04 (0.26) | 0.21 (0.49) | -0.34 (0.31) | -0.36 (0.29) |
| Peripheral arterial disease | 0.55 (0.17) | 0.13 (0.15) | 13.68 (444.20) | 0.09 (0.20) | 0.23 (0.22) |
| Carotid artery disease | 1.10 (0.21) | 0.67 (0.16) | -0.44 (0.65) | 0.27 (0.19) | 0.28 (0.23) |
| Cardiologist care | 0.28 (0.09) | 0.59 (0.15) | 0.16 (0.18) | -0.10 (0.19) | -0.14 (0.24) |
| Endocrinologist care | 0.08 (0.15) | 0.05 (0.15) | -0.27 (0.40) | -0.04 (0.19) | -0.20 (0.20) |
| Neurologist care | 0.17 (0.09) | -0.22 (0.14) | -0.09 (0.18) | 0.13 (0.14) | 0.02 (0.16) |
| Number of E&M visits | -0.01 (0.00) | -0.01 (0.00) | 0.00 (0.00) | 0.00 (0.00) | 0.00 (0.00) |
| Hospitalization for any cause | 0.11 (0.08) | -0.01 (0.11) | 0.35 (0.15) | -0.05 (0.15) | 0.19 (0.17) |
| Cardiac stress test | -0.06 (0.09) | -0.03 (0.11) | 0.62 (0.48) | -0.07 (0.15) | -0.11 (0.17) |
| Echocardiogram | -0.41 (0.24) | 0.00 (0.10) | -0.49 (0.18) | 0.20 (0.14) | 0.08 (0.16) |
| Electrocardiogram | -0.28 (0.09) | -0.48 (0.14) | -0.32 (0.17) | -0.15 (0.19) | -0.07 (0.24) |
| Interaction terms |  |  |  |  |  |
| Female × tobacco use | 0.41 (0.21) | NA | NA | NA | NA |
| Female × hyperlipidemia | 0.37 (0.13) | NA | 0.96 (0.31) | 0.48 (0.27) | NA |
| Female × diabetes | 0.64 (0.18) | NA | NA | NA | NA |
| Female × coronary revascularization | 12.78 (240.70) | 0.74 (0.25) | NA | NA | NA |
| Female × coronary heart disease | -1.11 (0.19) | NA | NA | NA | NA |
| Female × abdominal aortic aneurysm | NA | 1.16 (0.58) | NA | NA | NA |
| Female × peripheral arterial disease | NA | NA | -13.57 (444.20) | NA | NA |
| Female × neurologist care | NA | 0.37 (0.19) | NA | NA | NA |
| Female × cardiac stress test | NA | NA | -0.64 (0.37) | NA | NA |
| Female × echocardiogram | 0.25 (0.15) | NA | NA | NA | NA |

Abbreviations: E&M, evaluation and management; NA, not applicable.

* Numbers are beta coefficient (standard error).

† Area-level income quintiles: Quintile 1: <$23,967/y; Quintile 2: $23,967-$31,330/y; Quintile 3: $31,331-$39,175/y; Quintile 4: $39,176-$51,710/y; Quintile 5: ≥$51,711/y.

**Supplemental Table 6. Sensitivity analyses: Test characteristics of Medicare claims-based models using modified definitions for identifying high risk groups defined in REGARDS study data**

| **Condition and model*** | **N** | **Prevalence of condition** | **Predicted probability threshold** | **Sensitivity**  **(95% CI)** | **Specificity**  **(95% CI)** | **Positive predictive value**  **(95% CI)** | **Negative predictive value**  **(95% CI)** | **C statistic**  **(95% CI)** |
| --- | --- | --- | --- | --- | --- | --- | --- | --- |
| *Among all eligible participants* |  |  |  |  |  |  |  |  |
| Condition 1: High risk for CHD events,  Predicted probability assigned as 1 or 0^†^ | 6,615 | 49% |  |  |  |  |  |  |
| Pre-specified |  |  | --- | 0.75  (0.73, 0.77) | 0.83  (0.81, 0.84) | 0.80  (0.79, 0.81) | 0.78  (0.76, 0.79) | --- |
| Condition 1: High risk for CHD events,  Predicted probability assigned as 1 or model-based^‡^ | 6,615 | 49% |  |  |  |  |  |  |
| Pre-specified |  |  | 0.58 | 0.75  (0.73, 0.77) | 0.83  (0.82, 0.85) | 0.80  (0.79, 0.82) | 0.78  (0.77, 0.80) | 0.86  (0.85, 0.86) |
| Pre-specified + data mining |  |  | 0.71 | 0.75  (0.73, 0.76) | 0.83  (0.81, 0.84) | 0.80  (0.79, 0.81) | 0.77  (0.76, 0.79) | 0.87  (0.86,0.88) |
| Condition 1: Very high risk for CHD events,  Predicted probability assigned as 1 or model-based^§^ | 6,615 | 14% |  |  |  |  |  |  |
| Pre-specified |  |  | 0.26 | 0.63  (0.58, 0.66) | 0.90  (0.89, 0.91) | 0.51  (0.48, 0.54) | 0.94  (0.93, 0.95) | 0.84  (0.83, 0.86) |
| Pre-specified + data mining |  |  | 0.26 | 0.66  (0.65, 0.68) | 0.90  (0.87, 0.90) | 0.51  (0.50, 0.53) | 0.93  (0.90, 0.94) | 0.86  (0.84, 0.86) |

* Pre-specified Medicare variables: age, sex, race, Medicaid eligible, area-level income, geographic region, evidence of tobacco use, history of hyperlipidemia, history of hypertension, history of diabetes, acute MI, coronary revascularization, history of CHD, history of stroke, history of abdominal aortic aneurysm, history of peripheral arterial disease, history of carotid artery disease, cardiologist care, endocrinologist care, neurologist care, number of evaluation and management visits, hospitalization for any cause, cardiac stress test, echocardiogram, electrocardiogram. For pre-specified variable definitions and explanation of data mining variables see Supplemental Methods.

^†^ In this sensitivity analysis for identifying high risk for CHD events, we assigned predicted probability = 1 for each participant who met a pre-specified claims-based definition of high risk for CHD events, and assigned predicted probability = 0 otherwise.

^‡^ In this sensitivity analysis for identifying high risk for CHD events, we assigned predicted probability = 1 for each participant who met a pre-specified claims-based definition of high risk for CHD events, and assigned a predicted probability based on a logistic regression model otherwise. Test characteristics, corrected for optimism using bootstrap resampling, are reported for the predicted probability threshold corresponding to an uncorrected specificity of 0.83, which was the maximum uncorrected specificity achieved in both the pre-specified and the pre-specified plus data mining models.

^§^ In this sensitivity analysis for identifying very high risk for CHD events, we assigned predicted probability = 1 for each participant who met a pre-specified claims-based definition of very high risk for CHD events, and assigned a predicted probability based on a logistic regression model otherwise. Test characteristics, corrected for optimism using bootstrap resampling, are reported for the predicted probability threshold corresponding to an uncorrected specificity of 0.90.

**Supplemental Table 7. Sensitivity analyses: Test characteristics of Medicare claims-based models using one year of claims for identifying high risk groups defined in REGARDS study data**

| **Condition and model*** | **N** | **Prevalence of condition** | **Predicted probability threshold^†^** | **Sensitivity**  **(95% CI)^‡^** | **Specificity**  **(95% CI)^‡^** | **Positive predictive value**  **(95% CI)^‡^** | **Negative predictive value**  **(95% CI)^‡^** | **C statistic**  **(95% CI)^‡^** |
| --- | --- | --- | --- | --- | --- | --- | --- | --- |
| *Among all eligible participants* |  |  |  |  |  |  |  |  |
| Condition 1: High risk for CHD events | 6,615 | 49% |  |  |  |  |  |  |
| Pre-specified |  |  | 0.52 | 0.62  (0.60, 0.63) | 0.90  (0.89, 0.91) | 0.85  (0.83, 0.86) | 0.71  (0.69, 0.72) | 0.83  (0.82, 0.84) |
| Pre-specified + data mining |  |  | 0.54 | 0.66  (0.64, 0.67) | 0.89  (0.88, 0.90) | 0.85  (0.83, 0.86) | 0.73  (0.71, 0.74) | 0.84  (0.83, 0.85) |
| Condition 2: Very high risk for CHD events | 6,615 | 14% |  |  |  |  |  |  |
| Pre-specified |  |  | 0.25 | 0.53  (0.50, 0.56) | 0.90  (0.89, 0.91) | 0.47  (0.44, 0.50) | 0.92  (0.91, 0.93) | 0.78  (0.77, 0.80) |
| Pre-specified + data mining |  |  | 0.26 | 0.59  (0.56, 0.63) | 0.90  (0.89, 0.90) | 0.49  (0.46, 0.52) | 0.93  (0.92, 0.93) | 0.81  (0.80, 0.83) |
| *Among eligible participants without history of CHD or risk equivalents* |  |  |  |  |  |  |  |  |
| Condition 3: Framingham risk score >20% | 3,720 | 9% |  |  |  |  |  |  |
| Pre-specified |  |  | 0.20 | 0.41  (0.35, 0.46) | 0.90  (0.89, 0.91) | 0.28  (0.24, 0.32) | 0.94  (0.93, 0.95) | 0.80  (0.78, 0.82) |
| Pre-specified + data mining |  |  | 0.20 | 0.44  (0.38, 0.48) | 0.90  (0.89, 0.91) | 0.29  (0.25, 0.33) | 0.95  (0.93, 0.95) | 0.81  (0.79, 0.83) |

* Pre-specified Medicare variables: age, sex, race, Medicaid eligible, area-level income, geographic region, evidence of tobacco use, history of hyperlipidemia, history of hypertension, history of diabetes, acute MI, coronary revascularization, history of CHD, history of stroke, history of abdominal aortic aneurysm, history of peripheral arterial disease, history of carotid artery disease, cardiologist care, endocrinologist care, neurologist care, number of evaluation and management visits, hospitalization for any cause, cardiac stress test, echocardiogram, electrocardiogram. For pre-specified variable definitions and a description of the methods used to obtain variables through a data mining procedure see Supplemental Methods.

^†^ For each model, test characteristics are reported for the predicted probability threshold corresponding to an uncorrected specificity of 0.90.

^‡^ Corrected for optimism using bootstrap resampling.

**Supplemental Table 8. Sensitivity analyses: Test characteristics of Medicare claims-based models using one year of claims for identifying uncontrolled LDL cholesterol among statin users at high risk for CHD events defined in REGARDS study data**

| **Condition and model*** | **N** | **Prevalence of condition** | **Predicted probability threshold^†^** | **Sensitivity**  **(95% CI)^‡^** | **Specificity**  **(95% CI)^‡^** | **Positive predictive value**  **(95% CI)^‡^** | **Negative predictive value**  **(95% CI)^‡^** | **C statistic**  **(95% CI)^‡^** |
| --- | --- | --- | --- | --- | --- | --- | --- | --- |
| *Among eligible participants at high risk for CHD events who were using statins* |  |  |  |  |  |  |  |  |
| Condition 4: LDL cholesterol ≥100 mg/dL | 1,583 | 30% |  |  |  |  |  |  |
| Pre-specified |  |  | 0.43 | 0.18  (0.15, 0.21) | 0.89  (0.87, 0.90) | 0.43  (0.38, 0.48) | 0.71  (0.69, 0.73) | 0.59  (0.56, 0.61) |
| Pre-specified + data mining |  |  | 0.44 | 0.22  (0.18, 0.24) | 0.88  (0.86, 0.90) | 0.46  (0.41, 0.51) | 0.72  (0.70, 0.75) | 0.61  (0.58, 0.63) |
| Condition 5: LDL cholesterol ≥70 mg/dL | 1,583 | 80% |  |  |  |  |  |  |
| Pre-specified |  |  | 0.87 | 0.22  (0.20, 0.25) | 0.86  (0.82, 0.90) | 0.88  (0.86, 0.92) | 0.22  (0.20, 0.24) | 0.59  (0.56, 0.62) |
| Pre-specified + data mining |  |  | 0.88 | 0.25  (0.22, 0.27) | 0.85  (0.80, 0.89) | 0.88  (0.85, 0.91) | 0.22  (0.19, 0.23) | 0.62  (0.59, 0.65) |

* Pre-specified Medicare variables: age, sex, race, Medicaid eligible, area-level income, geographic region, evidence of tobacco use, history of hyperlipidemia, history of hypertension, history of diabetes, acute MI, coronary revascularization, history of CHD, history of stroke, history of abdominal aortic aneurysm, history of peripheral arterial disease, history of carotid artery disease, cardiologist care, endocrinologist care, neurologist care, number of evaluation and management visits, hospitalization for any cause, cardiac stress test, echocardiogram, electrocardiogram. For pre-specified variable definitions and a description of the methods used to obtain variables through a data mining procedure see Supplemental Methods.

^†^ For each model, test characteristics are reported for the predicted probability threshold corresponding to an uncorrected specificity of 0.90.

^‡^ Corrected for optimism using bootstrap resampling.
